# Initiation of Experimental Temporal Lobe Epilepsy by Early Astrocyte Uncoupling Is Independent of TGFβR1/ALK5 Signaling

**DOI:** 10.3389/fneur.2021.660591

**Published:** 2021-05-07

**Authors:** Lukas Henning, Christian Steinhäuser, Peter Bedner

**Affiliations:** Institute of Cellular Neurosciences, Medical Faculty, University of Bonn, Bonn, Germany

**Keywords:** blood–brain barrier dysfunction, albumin extravasation, temporal lobe epilepsy, gap junctional coupling, astrocyte, transforming growth factor beta

## Abstract

Blood–brain barrier (BBB) dysfunction following brain insults has been associated with the development and progression of focal epilepsy, although the underlying molecular mechanisms are not fully elucidated yet. Activation of transforming growth factor beta (TGFβ) signaling in astrocytes by extravasated albumin impairs the ability of astrocytes to properly interact with neurons, eventually leading to epileptiform activity. We used the unilateral intracortical kainate mouse model of temporal lobe epilepsy (TLE) with hippocampal sclerosis (HS) to gain further insights into the role of BBB leakage in *status epilepticus* (SE)-induced epileptogenesis. Immunohistochemical examination revealed pronounced albumin extravasation already 4 h after SE induction. Astrocytes were virtually devoid of albumin immunoreactivity (IR), indicating the lack of uptake by this time point. Inhibition of the TGFβ pathway by the specific TGFβ receptor 1 (TGFβR1) kinase inhibitor IPW-5371 did not prevent seizure-induced reduction of astrocytic gap junction coupling. Thus, loss of coupling, which is thought to play a causative role in triggering TLE-HS, is most likely not mediated by extravasated albumin. Continuous telemetric EEG recordings and video monitoring performed over a period of 4 weeks after epilepsy induction revealed that inhibition of the TGFβ pathway during the initial phase of epileptogenesis slightly attenuated acute and chronic epileptiform activity, but did not reduce the extent of HS. Together, these data indicate that albumin extravasation due to increased BBB permeability and TGFβ pathway activation during the first hours after SE induction are not significantly involved in initiating TLE.

## Key Points

- Strong albumin extravasation, but no astrocytic uptake, was detected 4 h after epilepsy induction.
- Seizure-induced reduction of astrocytic gap junction coupling was independent of TGFβR1/ALK5 signaling.
- Inhibition of TGFβR1/ALK5 signaling during early epileptogenesis slightly attenuated epileptiform activity but did not prevent the development of hippocampal sclerosis in experimental temporal lobe epilepsy.

## Introduction

Epilepsy is often preceded by epileptogenic brain insults including traumatic brain injury, febrile seizures, or stroke (1–3). Impairment in blood–brain barrier (BBB) integrity is often associated with these insults and also occurs in the sclerotic hippocampus of patients with temporal lobe epilepsy (TLE) and corresponding animal models (4–7). Blood–brain barrier leakage leads to extravasation of the serum protein albumin into the brain parenchyma, which promotes epileptiform activity both *in situ* and *in vivo* (6, 8–10). Interestingly, serum albumin was proposed to be endocytosed into astrocytes *via* binding to transforming growth factor beta (TGFβ) receptors and to activate TGFβ signaling pathways (5, 6, 8). Albumin-induced TGFβ signaling in astrocytes in turn provokes several physiological changes in these cells associated with the development of epilepsy, including excitatory synaptogenesis, impaired K^+^ and glutamate buffering, and production of pro-inflammatory cytokines (6, 11–13). Previously, we could demonstrate that disruption of astrocytic gap junction coupling and concomitant impairment of extracellular K^+^ buffering precede neuronal cell death in kainate-induced TLE, pointing toward a causative role in epileptogenesis (14). However, the underlying signaling pathway remains ill-defined. Interestingly, our previous work showed that albumin injected into the lateral ventricle is transported into hippocampal astrocytes leading to impaired astrocytic gap junction coupling 24 h after albumin injection (15). This was further supported by other findings demonstrating significant downregulation of transcripts for both astroglial gap junction-forming connexins, 24 h after albumin treatment in the rat brain (5, 11, 16). This suggests that albumin uptake into astrocytes promotes gap junction uncoupling *via* activation of TGFβ signaling, leading to impaired spatial buffering of extracellular K^+^ and thereby promoting epileptic activity. To test this hypothesis, here we assessed in a mouse model of TLE whether aberrant albumin-mediated TGFβR1/ALK5 signaling upon kainate-induced *status epilepticus* (SE) induces astrocyte uncoupling and epileptogenesis. We took advantage of a novel TGFβR1 kinase inhibitor, IPW-5371, which effectively reduced TGFβ signaling in the hippocampus of mice receiving intraventricular infusion of albumin (17). Immunohistochemistry, tracer diffusion studies, and continuous telemetric electroencephalography (EEG) were combined to assess the consequences of early inhibition of TGFβR1/ALK5 signaling on astrocytic coupling, seizure activity, and the development of HS.

## Materials and Methods

### Animals

Male C57B6/J (Charles River, Sulzfeld, Germany, or bred in-house) mice aged 90–120 days were used for the experiments. Maintenance and handling of animals was performed according to local governmental regulations. Experiments were approved by the North Rhine–Westphalia State Agency for Nature, Environment and Consumer Protection (approval number 84-02.04.2015.A393). All measures were taken to minimize the number of animals used. Mice were kept under standard housing conditions (12 h/12 h dark–light cycle) with food and water provided *ad libitum*.

### Unilateral Intracortical Kainate Injection and Implantation of Telemetric Electroencephalography Transmitters

We employed the TLE-HS animal model as described previously (13, 18). Briefly, mice were anesthetized with a mixture of medetomidine (Cepetor, CP-Pharma, Burgdorf, Germany, 0.3 mg/kg, i.p.) and ketamine (Ketamidor, WDT, Garbsen, Germany, 40 mg/kg, i.p.) and placed into a stereotaxic frame equipped with a manual microinjection unit (TSE Systems GmbH, Bad Homburg, Germany). A total volume of 70 nl of a 20-mM solution of kainate (Tocris, Bristol, UK) in 0.9% sterile NaCl was stereotactically injected into the neocortex just above the right dorsal hippocampus. The stereotactic coordinates were 2 mm posterior to bregma, 1.5 mm from midline, and 1.7 mm from the skull surface. Sham control mice received injections of 70 nl saline under the same conditions. Directly after kainate injection, two drill holes were made at 1 mm posterior to the injection site and 1.5 mm lateral from midline for insertion of two monopolar leads required for electrographic seizure detection. Telemetric transmitters [TA10EA-F20 or TA11ETA-F10; Data Sciences International (DSI), St. Paul, MN, USA] were implanted subcutaneously into the right abdominal region, and both monopolar leads were inserted ~1 mm into the cortex. Attached leads were fixed to the skull using superglue and then covered with dental cement. Subsequently, the scalp incision was sutured and anesthesia stopped with atipamezol (Antisedan, Orion Pharma, Hamburg, Germany, 300 mg/kg, i.p.). To reduce pain, mice were injected for 3 days with carprofen (Rimadyl, Pfizer, Karlsruhe, Germany). Moreover, 0.25% enrofloxacin (Baytril, Bayer, Leverkusen, Germany) was administered *via* drinking water to reduce risk of infection. After surgery, mice were returned to clean cages and placed on individual radio receiving plates (RPC-1; Data Sciences International, New Brighton, MN, USA), which capture data signals from the transmitter and send them to a computer using the Ponemah software (Version 5.2, Data Sciences International) to convert the digital output of the receiver into a calibrated analog output. A video surveillance system (Bascom, Düsseldorf, Germany) was used to monitor behavioral seizure activity. Electroencephalography recordings (24 h/day, 7 days/week) were started immediately after transmitter implantation and continued for 28 days following the induction of SE.

### Electroencephalography Analysis

Electroencephalography data were analyzed using NeuroScore (version 3.3.1) software (Data Sciences International) as described previously (19). Briefly, seizure frequency and duration as well as spike numbers was determined using the spike train analysis tool implemented in NeuroScore with the following criteria: threshold value = 7.5 × SD of the baseline (i.e., activity during artifact- and epileptiform-free epochs) – 1,000 μV, spike duration = 0.1–50 ms, spike interval = 0.1–2.5 s, minimum train duration = 30 s, train join interval = 1 s, and minimum number of spikes = 50. Prior to spike analysis, recordings were high pass filtered at 1 Hz. Electroencephalography recordings were additionally verified by manual screening. Fast Fourier transformation (FFT) was performed to derive absolute δ (0.5–4 Hz), θ (4–8 Hz), α (8–13 Hz), β (13–30 Hz), and γ (30–50 Hz) power values during SE and the chronic phase, which were subsequently normalized to baseline activity prior to conducting statistics. The number of spontaneously generalized seizures during the chronic phase was determined manually by two experienced experimenters.

### IPW-5371 Treatment

IPW-5371 was prepared as a suspension formulation (2 mg/ml IPW-5371 in 0.5% methylcellulose dissolved in 0.9% NaCl and Tween® 80) and applied once per 16–24 h and again ~15 min prior to kainate application (i.p. injection, 20 mg/kg). Daily i.p. injections of IPW-5371 at 20 mg/kg for 2 days effectively reduce TGFβ signaling in the hippocampus of mice receiving intraventricular infusion of albumin (17). As a control, mice received vehicle (saline) injections under the same conditions.

### Electrophysiology and Biocytin Loading of Astrocytes

Mice were anesthetized with isoflurane (Piramal Healthcare, Morpeth, UK) and decapitated. Next, brains were quickly removed and 200-μm-thick coronal slices were cut on a vibratome (VT1000S, Leica, Wetzlar, Germany) in an ice-cold preparation solution containing the following (in mM): 87 NaCl, 2.5 KCl, 1.25 NaH_2_PO_4_, 25 NaHCO_3_, 7 MgCl_2_, 0.5 CaCl_2_, 25 glucose, and 75 sucrose, equilibrated with carbogen (5% CO_2_/95% O_2_, pH 7.4). After storage of slices (15 min, 35°C) in preparation solution, the slices were transferred to a solution containing the following (in mM): 126 NaCl, 3 KCl, 2 MgSO_4_, 2 CaCl_2_, 10 glucose, 1.25 NaH_2_PO_4_, and 26 NaHCO_3_ and gassed with carbogen to stabilize pH at 7.4 [artificial cerebrospinal fluid (aCSF)]. To aid in the identification of astrocytes in the tissue, aCSF was supplemented with SR101 (1 μM, Sigma Aldrich, S7635, Steinheim, Germany; incubation 20 min, 35°C) (20). After SR101 staining, slices were transferred to aCSF and kept at room temperature (RT) for the duration of the experiments. For recordings, slices were transferred to a recording chamber and constantly perfused with aCSF. Patch pipettes fabricated from borosilicate capillaries with a resistance of 3–6 MΩ were filled with a solution containing the following (in mM): 130 K-gluconate, 1 MgCl_2_, 3 Na_2_-ATP, 20 HEPES, 10 EGTA, and biocytin (0.5%, Sigma Aldrich) (pH 7.2, 280–285 mOsm). For the analysis of gap junction coupling, whole-cell patch clamp recordings of SR101-positive astrocytes were performed during which astrocytes were filled with biocytin (20 min, RT). In addition to SR101 staining, astrocytes were identified by their characteristic morphology, small soma size, passive current–voltage relationship, and a resting membrane potential close to the Nernst potential for K^+^. Current signals were amplified (EPC 8, HEKA Electronic, Lambrecht, Germany), filtered at 3 or 10 kHz, and sampled at 10 or 30 kHz (holding potential −70 mV). Online analysis was performed with TIDA 5.25 acquisition and analysis software for Windows (HEKA) and Igor Pro 6.37 software (WaveMetrics, Lake Oswego, OR, USA). Voltages were corrected for liquid junction potentials. Only recordings matching the following criteria were included in the analysis: (i) resting potential negative to −60 mV, (ii) membrane resistance ≤ 10 MΩ, and (iii) series resistance ≤ 20 MΩ.

### Immunohistochemistry

#### Tissue Preparation

Animals were deeply anesthetized by i.p. injection with 100–120 μl of a solution containing 80 mg/kg ketamine (Ketamidor, WDT, Garbsen, Germany) and 1.2 mg/kg xylazine hydrochloride (Sigma-Aldrich). After checking for hind paw reflexes, transcardial perfusion was applied with ice-cold PBS (30 ml) followed by 4% ice-cold PFA in PBS (30 ml). Brains were removed and stored overnight in 4% PFA-containing solution and subsequently stored in PBS at 4°C until slicing. Brains were cut into 40-μm-thick coronal slices using a Leica VT1200S vibratome (Leica Microsystems).

#### Staining

Immunohistochemistry was performed using free-floating slices kept in 24-well plates. Only slices from the dorsal hippocampus close to the injection site were used for staining. For membrane permeabilization and blocking of unspecific epitopes, slices were incubated (2 h, RT) with 0.5% Triton X-100 (or 2% for staining of biocytin-filled astrocytes) and 10% normal goat serum (NGS) in PBS. For immunostaining of albumin, no serum was applied during blocking and permeabilization steps. Slices were subsequently incubated overnight with primary antibody solution containing PBS on a shaker at 4°C. The following primary antibodies were applied: rabbit anti-GFAP (1:500, DAKO, Z0334, Hamburg, Germany), goat anti-albumin (1:200, Abcam, ab19194, Berlin, Germany), and mouse anti-NeuN (1:200, Merck Millipore, MAB377, Darmstadt, Germany). On the following day, slices were washed three times with PBS for 10 min each, followed by incubation with secondary antibodies conjugated with Alexa Fluor® 488, Alexa Fluor® 647, or streptavidin-conjugated Alexa Fluor® 647 (1:500 or 1:600, respectively, Invitrogen, Karlsruhe, Germany) in PBS (2% NGS, 1.5–2 h, RT). For staining of NeuN, slices were incubated with goat anti-mouse biotin (1:500, Dianova, AB_2338557, Hamburg, Germany) prior to incubation with streptavidin-conjugated Cy3 antibody (1:300, Sigma Aldrich, S6402; 1 h, RT). After washing the slices again three times with PBS (10 min), nuclear staining with Hoechst (1:200, diluted in dH_2_O) was performed (10 min, RT). A final washing step (3 × PBS, 5 min each) was performed and slices were mounted with Aqua-Poly/Mount (Polysciences, Heidelberg, Germany) on objective slides and covered with coverslips. Slides were stored at 4°C before confocal imaging.

#### Confocal Microscopy

Slides were imaged using a confocal laser scanning microscope (SP8, Leica, Hamburg, Germany) at 8 bit using 20 × [numerical aperture (NA): 0.75], 40 × (NA: 1.1), and 63 × (NA: 1.2) objectives. Image resolution was set at 1,024 × 1,024 pixels recorded at a speed of 400 Hz, with a pinhole size of 1 airy unit (AU) and a digital zoom of 1 (albumin extravasation), 1.2 (GJ coupling), or 2 (colocalization). Standard photomultiplier tubes were used for the detection of fluorescent signals. Laser and detector settings were applied equally to all images acquired. Z-stacks were taken at 2 μm (albumin extravasation and GJ coupling) or 0.3 μm (colocalization) intervals.

### Quantification of Immunostainings

Immunohistochemical stainings were quantified either using Fiji/ImageJ (21) or Imaris 8.0 software (Bitplane, Zürich, Switzerland).

#### Albumin Extravasation

Albumin extravasation in the parenchyma was estimated by measuring the fluorescent intensity in the albumin channel in maximum intensity projections (MIP) using the image processing package Fiji. Initially, images were background subtracted using the rolling ball algorithm implemented in Fiji, with a radius set at 50 pixels (22). Albumin immunoreactivity (IR) was subsequently determined by quantifying the average pixel intensity in the albumin channel within the imaged field of view (290 × 290 × 20 μm).

#### Colocalization of Albumin and GFAP

Albumin content was quantified in albumin/GFAP double-stained hippocampal sections of C57B6J mice injected with kainate 4 h prior to brain perfusion. Images were analyzed equally applying a custom-written macro in Fiji software. Fluorescent intensity analysis was performed in regions of interest (ROIs) of 92 × 92 × 10 μm3. In a first step, the GFAP^+^ image was median filtered (5 pixel radius) and subsequently converted into a binary image applying the Triangle threshold algorithm implemented in Fiji (23). Next, the binary GFAP^+^ images and the albumin^+^ images were multiplied to derive albumin signal intensity in GFAP^+^ pixels. Within this multiplied image, the average pixel intensity value of albumin in GFAP^+^ pixels was measured and subsequently summed up across all focal plains to obtain albumin contents in GFAP^+^ cells. To determine albumin content in GFAP^−^ pixels, the GFAP image was preprocessed applying filtering and thresholding steps as described above. Next, the binarized GFAP^+^ image was subtracted from the albumin^+^ image to obtain albumin signal intensity in GFAP^−^ pixels. Average signal intensities were subsequently summed up across all focal plains for statistical comparison.

#### Coupling Efficiency in Biocytin-Filled Astrocytes

Coupling efficiency was determined by manual counting of biocytin^+^ cells using the cell counter plugin for Fiji and compared between injected (ipsilateral) and non-injected (contralateral) hemispheres. Another observer blinded to the experimental conditions recounted images of biocytin-filled astrocytes, and cell counts were subsequently averaged across both counts prior to statistical analysis.

#### Hippocampal Sclerosis

The extent of hippocampal sclerosis (HS) was estimated based on the quantification of three parameters: (i) extent of granule cell dispersion (GCD) in the dentate gyrus (DG), (ii) shrinkage of the CA1 *stratum radiatum*, and (iii) number of pyramidal neurons in CA1 *stratum radiatum*. All three parameters were estimated in MIPs (1,163 × 1,163 × 40 μm3). Granule cell dispersion quantification was performed as described previously (19). Briefly, GCL width was measured at four positions indicated as T1–T4. T1 and T2 were measured along a vertical line connecting the upper and lower cell layers of the DG, T3, and T4 at a distance halfway between the vertical line and the tip of the hilus. The average of the four values was used as an estimation of GCD. Shrinkage of the *stratum radiatum* was determined by drawing a vertical line connecting the pyramidal and molecular layer, above the peak of the DG granule cell layer. The length of the vertical line served as an indication of the remaining width of the *stratum radiatum*. Both GCL and *stratum radiatum* width were quantified using Fiji software. Finally, the number of pyramidal neurons in the CA1 region was quantified using the automated spot detection algorithm implemented in Imaris 8.0 within a 360 × 120 × 40-μm^3^ ROI placed within the CA1 pyramidal layer just above the peak of the DG granule cell layer.

### Statistical Analysis

Statistical analyses were performed using R software (R Core Team 2020, version 4.0.2, Austria) (24). Data are displayed as mean ± SD or as box plots representing median (line) and quartiles (25^th^ and 75^th^ percentile) with whiskers extending to the highest and lowest values within 1.5 times the interquartile range (IQR). Prior to statistical analysis, data were checked for normality by inspection of histograms as well as by statistically testing for normality using a Shapiro–Wilk test. Levene's test was performed to check for homogeneity of variance between groups. In case of a significant deviation from normality, data were transformed according to Tukey's ladder of powers (25) prior to conduction of statistical tests or by performing the appropriate non-parametric test. For comparison of two groups, Student's *t*-test or Wilcoxon-rank sum test was used. More than two groups were compared with one-way analysis of variance (ANOVA) followed by *post-hoc* Tukey test or using Kruskal–Wallis test with Dunn's *post-hoc* test. For multifactorial data, two-way ANOVA was conducted. Kaplan–Meier estimates were compared using a log-rank test. Differences between means were considered significant at *p* < 0.05.

## Results

### Strong Albumin Extravasation but Negligible Astrocytic Uptake 4 h After *status epilepticus* Induction

**Figure 1:**
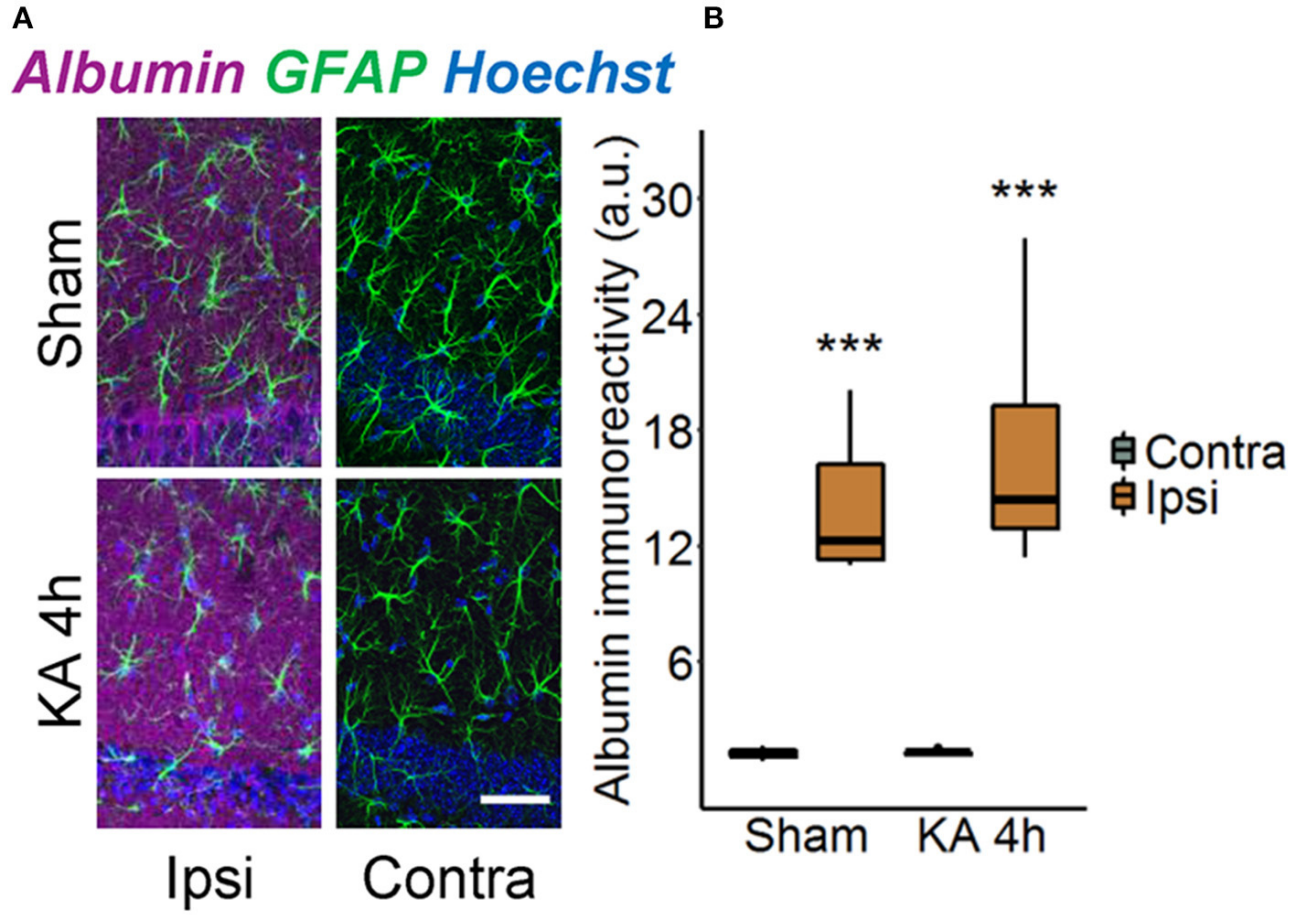
Ipsilateral albumin extravasation 4 h after *status epilepticus* (SE) induction. **(A)** Representative maximum intensity projections depicting albumin (magenta), GFAP (green), and Hoechst (blue) labeling in ipsi- and contralateral hippocampal slices of sham- and kainate-injected mice. Scale bar: 50 μm. **(B)** Quantification of the staining revealed substantially higher ipsilateral vs. contralateral albumin extravasation after injection of both kainate and saline. Box plots represent median and quartiles. ***p < 0.001 vs. contralateral (two-way ANOVA). *N* = 9 slices from three mice/group. KA, kainate; au, arbitrary unit.

Disruption of the BBB accompanied by albumin extravasation occurs in human and experimental epilepsy, and there is growing evidence that this represents not only a pathological consequence but also a causative factor in epileptogenesis (26, 27). To shed further light on this important topic, we used an experimental mouse model, unilateral intracortical kainate injection, that closely mimics human TLE-HS in terms of seizure types, neuropathological changes, and pattern of epileptogenesis (14). In this model, we first examined the extent of albumin extravasation 4 h after kainate injection, a time point preceding neuronal cell death and the onset of spontaneous seizure activity (14). Immunohistochemical staining revealed strong extravascular albumin IR in the hippocampal CA1 region of the dorsal ipsilateral hippocampus (Figure 1, *p* < 0.001, two-way ANOVA). On the contralateral side, albumin was largely confined to blood vessels, indicating that BBB breakdown was restricted to the ipsilateral hippocampus. Surprisingly, we also detected a significant increase of albumin IR in the hippocampus of sham-injected mice. However, whereas albumin extravasation in epileptic mice persisted for months (26), it was a transient event in sham-injected mice and disappeared within 5 days after injection (data not shown).

**Figure 2:**
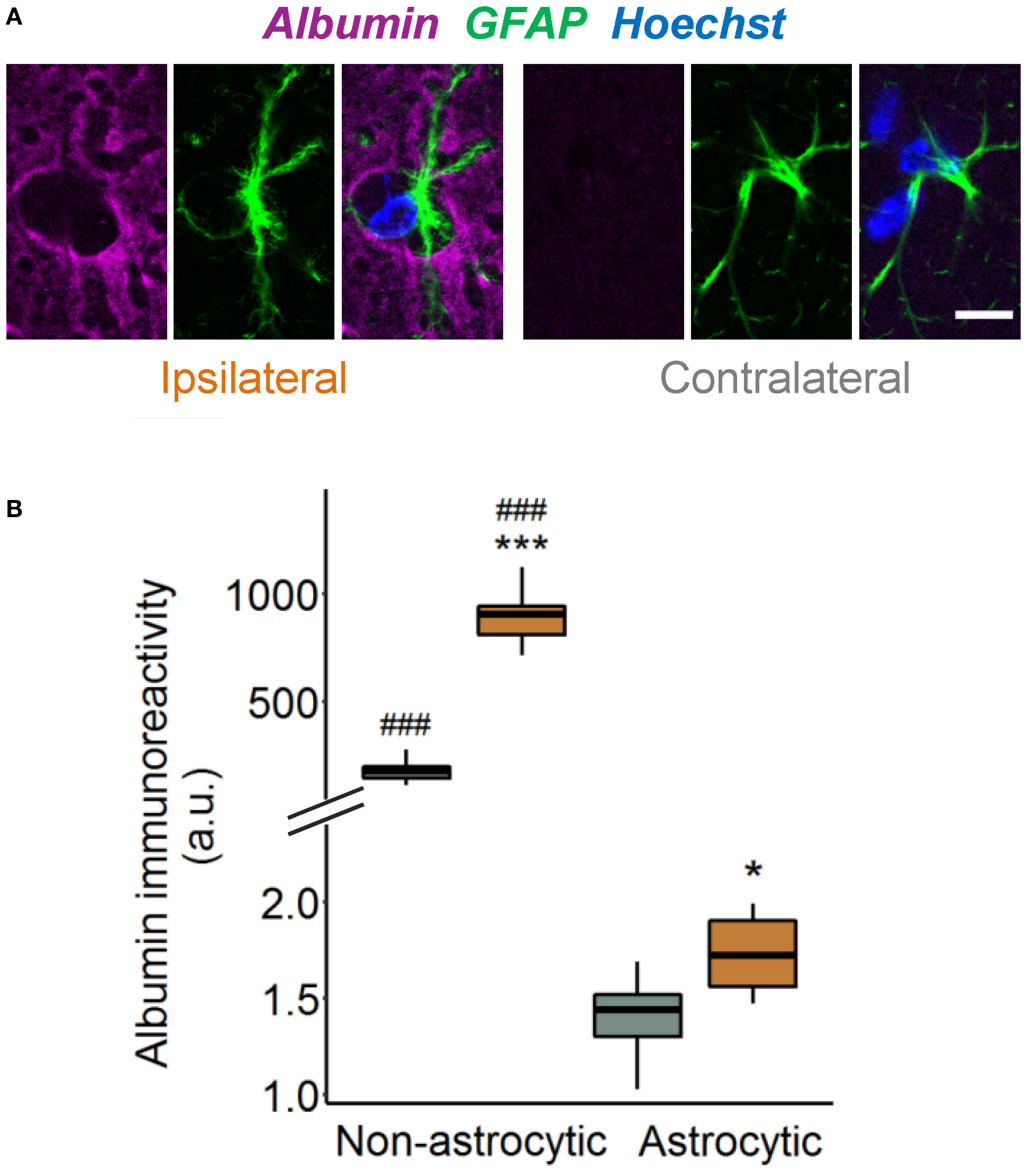
Tiny astrocytic albumin uptake 4 h after KA injection. **(A)** Representative images depicting one focal plane of combined albumin (magenta), GFAP (green), and Hoechst (blue) staining in the hippocampal CA1 *stratum radiatum* of ipsi- and contralateral slices, 4 h after kainate injection. For the purpose of illustration, images are background subtracted and adjusted for brightness. Scale bar: 10 μm. **(B)** Albumin immunoreactivity increases in both GFAP-positive and GFAP-negative pixels on the ipsi- vs. contralateral hemisphere, although the increase in astrocytes is orders of magnitude lower than outside the cells. Box plots represent median and quartiles. *p < 0.05, ***p < 0.001 vs. contralateral, *###p* < 0.001 vs. astrocytic (two-way ANOVA). *N* = 9 slices from three mice. au, arbitrary unit.

Previous works demonstrated that TGFβ receptor-mediated uptake of extravasated serum albumin into astrocytes is involved in epileptogenesis (6, 16). We therefore examined astrocytic albumin contents in albumin/GFAP double-stained hippocampal sections injected with kainate 4 h before (Figure 2A). Quantification revealed only faint albumin IR (<1%) in GFAP-positive compared with GFAP-negative pixels in the hippocampal CA1 region of both hemispheres (Figure 2B), indicating negligible astrocytic albumin uptake even in regions of high extravasation. Albumin IR was slightly higher in ipsilateral compared with contralateral astrocytes (Figure 2B, *p* = 0.016, two-way ANOVA), but because this effect was extremely small, its biological relevance is unclear. In conclusion, these results show that 4 h after epilepsy induction, uptake of extravasated albumin by astrocytes is negligible.

### Seizure-Induced Disruption of Astrocytic Coupling Is Independent of TGFßR1 Signaling

**Figure 3:**
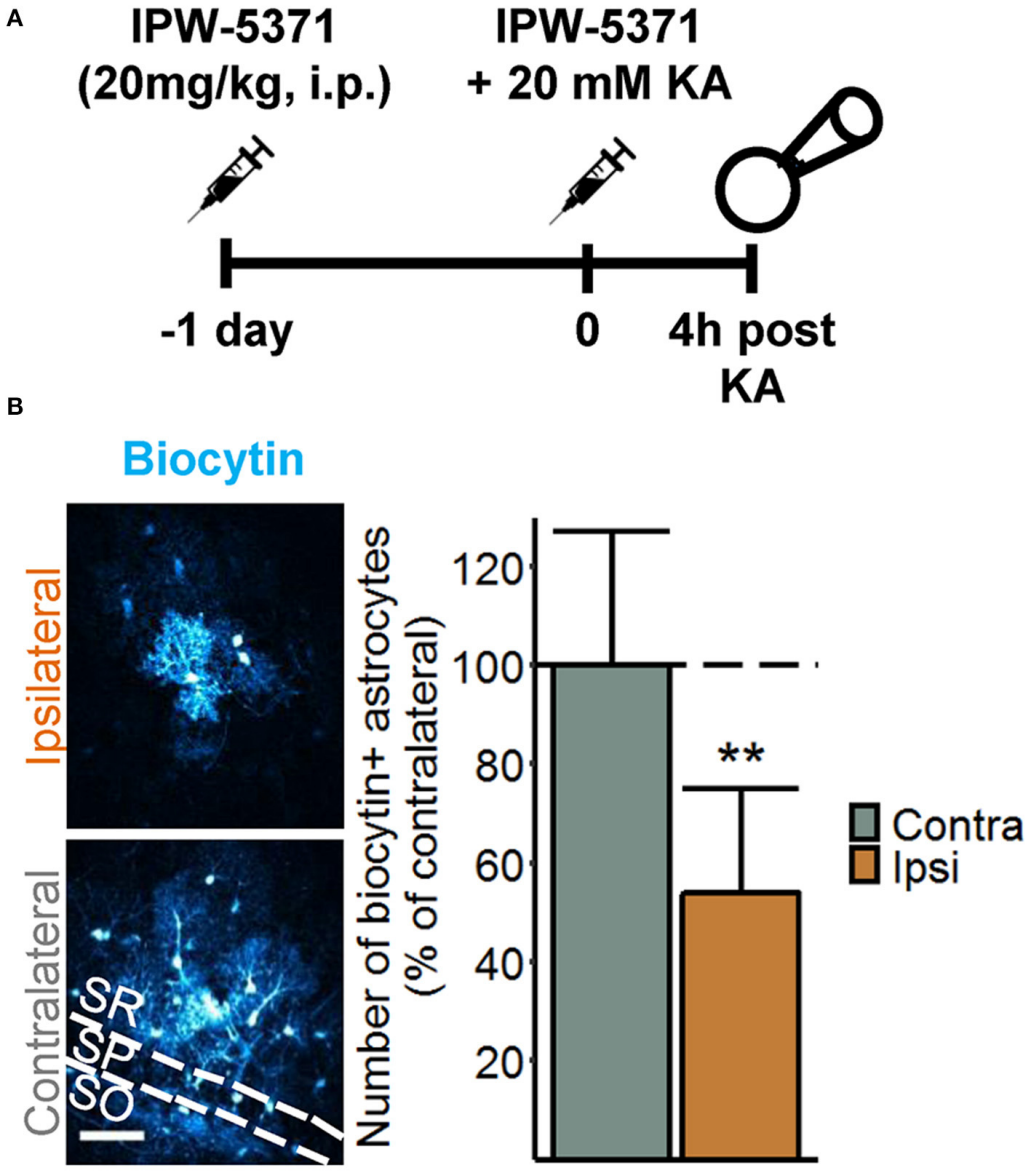
Seizure-induced loss of astrocytic gap junctional communication is not mediated by TGFßR1 signaling. **(A)** Injection scheme for the biocytin diffusion studies. IPW-5371 was injected intraperitoneally (i.p.) once per day at 20 mg/kg for two consecutive days prior to SE induction *via* intracortical kainate injection. Four hours after kainate injection, animals were sacrificed and gap junctional coupling between hippocampal astrocytes was visualized by the intercellular spread of biocytin, which was included in the patch pipette solution during whole-cell patch clamp recordings (20 min). **(B)** Representative maximum intensity projections depicting biocytin-filled astrocytes labeled with streptavidin-conjugated AlexaFluor® (AF) 647 in hippocampal CA1 *stratum radiatum* of the ipsi- and contralateral hemispheres (*left*). Scale bar: 50 μm. The number of biocytin-positive astrocytes was significantly reduced on the ipsi- vs. contralateral hippocampus of IPW-5371-pretreated mice (*right*). Data represent mean ± SD. **p < 0.01 (independent samples *t*-test). *N* = 9 slices/condition from three mice. KA, kainate; SR, *stratum radiatum*; SP, *stratum pyramidale*; SO, *stratum oriens*.

Disruption of astrocytic gap junctional communication is a characteristic feature of the sclerotic hippocampus of TLE patients and animal models. This astrocytic dysfunction and the consequential accumulation of extracellular K^+^ was detected already 4 h after intracortical kainate injection, leading to the suggestion that it plays a causative role in the pathogenesis of TLE (14). In another study, we demonstrated that intracerebroventricularly injected albumin is taken up by astrocytes and reduces their gap junctional coupling (15). Therefore, despite negligible astrocytic albumin uptake, the question arose whether activation of TGFβR1 by extravasated albumin mediates astrocyte uncoupling at this early stage of epileptogenesis. To address this question, we used a specific TGFβR1/ALK5 kinase inhibitor, IPW-5371, which has been shown to cross the BBB and to effectively reduce TGFβ signaling (17). IPW-5371 was injected i.p. (20 mg/kg) 1 day and 15 min prior to kainate application, and gap junction coupling was assessed 4 h later by biocytin filling of individual hippocampal astrocytes. The results show that IPW-5371 pretreatment did not prevent seizure-induced astrocyte uncoupling (Figure 3). Indeed, the number of biocytin-positive cells was reduced by 45% in the hippocampus of the injected hemisphere (ipsi 70 ± 26 vs. contra 128 ± 34 cells, mean ± SD, *p* = 0.0015, independent samples *t*-test), which corresponds to data from epileptic mice with undisturbed TGFβR1 signaling (14). Thus, TGFβ signaling appears not to be responsible for, or involved in, astrocyte uncoupling in experimental TLE.

### IPW-5371 Pretreatment Slightly Attenuates Acute and Chronic Epileptiform Activity but Has no Effect on the Development of HS in Experimental TLE

**Figure 4:**
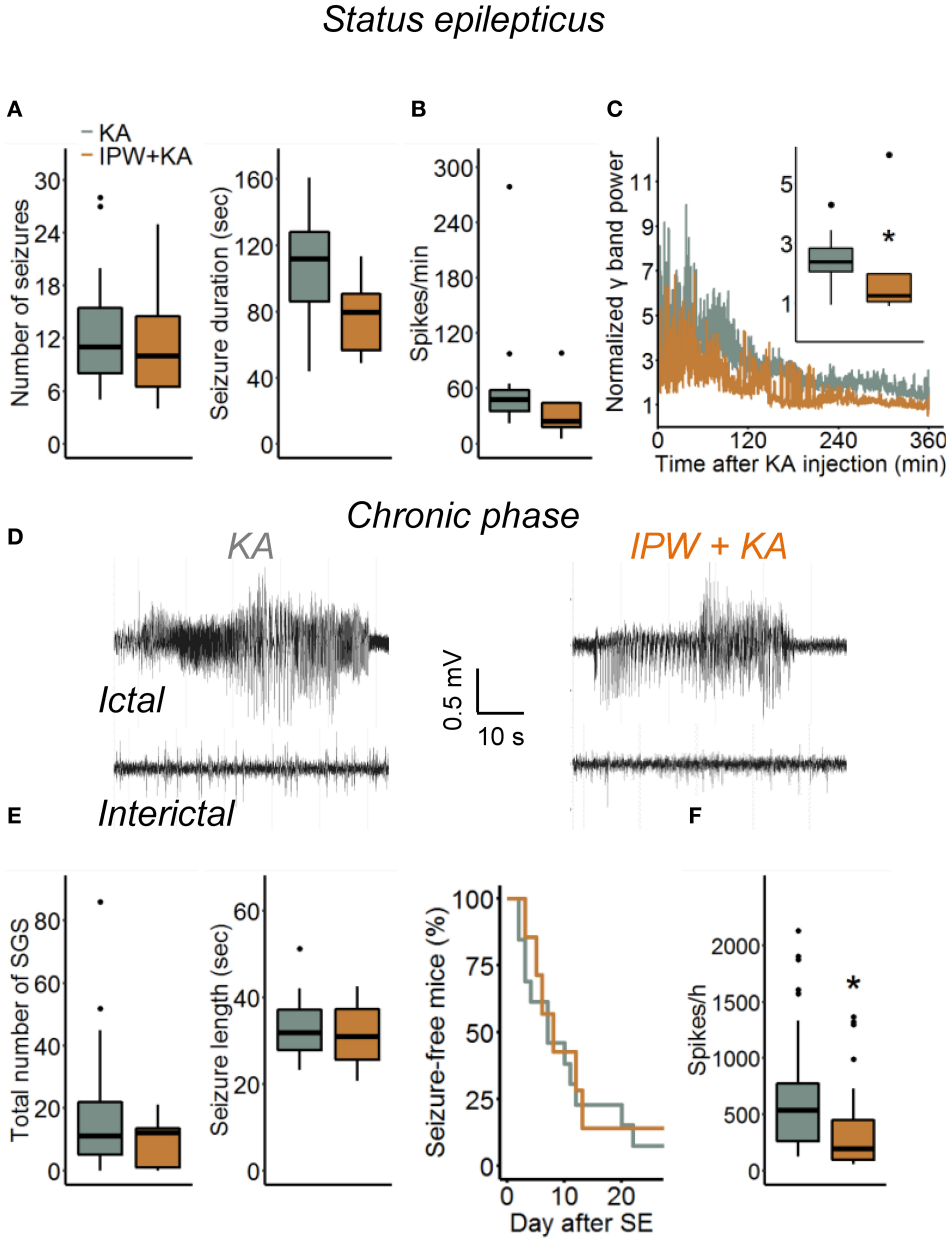
Kainate-induced acute and chronic epileptiform activity in IPW-5371-pretreated mice. **(A)** Number and duration of seizures during the first hour of kainate-induced SE did not differ between IPW-5371- and vehicle-treated kainate mice. **(B)** Severity of SE determined by counting the number of EEG spikes exceeding 7.5-fold baseline activity during the first 6 h after kainate injection. Spike activity was not affected by IPW-5371. **(C)** Spectral analysis yielded significantly lower γ band power in IPW-5371- vs. vehicle-treated kainate mice. **(D)** Representative EEG traces depicting spontaneous generalized seizures (*ictal*) and interictal spiking activity (*interictal*) in IPW-5371-treated and control mice. **(E)** Total number and length of spontaneous generalized seizures (SGS) in IPW-5371- and vehicle-treated kainate mice. Neither the number nor the length of SGS was significantly different between the experimental groups (left graph). The length of the latent phase was also similar in both groups (right graph). **(F)** The number of spikes/hour during the entire 4 weeks of recording was slightly but significantly reduced in IPW-5371- vs. vehicle-treated kainate mice. Box plots represent median and quartiles. *p < 0.05 (independent samples *t*-test). *N* = 7 (kainate + IPW-5371) and 13 (kainate) mice. KA, kainate.

Blood–brain barrier breakdown is implicated in the development of epilepsy through a mechanism involving astrocytic TGFβR1/ALK5 signaling (6, 9, 16, 28). To gain a deeper insight into this relationship, we investigated consequences of TGFβR1 kinase inhibition during the initial phase of epileptogenesis on acute and chronic electrographic epileptiform activity and histopathological changes in our TLE model. Mice were injected with IPW-5371 as described above and implanted with telemetric transmitters directly after kainate injection. Electroencephalography recording and video monitoring were subsequently performed continuously (24 h/day) over a period of 4 weeks. Quantification of SE, which in our model is manifested by a series of convulsive seizures lasting up to 6 h, was performed in three different ways: (a) by examining the number and duration of seizures and time spent in ictal activity during the first hour of recording, (b) by counting the number of EEG spikes with amplitudes exceeding baseline activity at least 7.5-fold during the first 6 h of recording, and (c) by comparison of the spectral power in the γ range after FFT of the EEG data. Notably, activity during epileptiform-free periods did not differ between experimental groups (γ power baseline: kainate 2.72 ± 1.36 nV^2^ vs. IPW + kainate 3.38 ± 0.97 nV^2^, *p* = 0.29, independent samples *t*-test; 7.5 × SD of baseline: kainate 196.61 ± 37.65 vs. IPW + kainate 222.75 ± 31.6, *p* = 0.136, independent samples *t*-test). We found no differences between kainate mice treated with IPW-5371 or vehicle regarding the number of seizures or seizure duration within the first hour of SE (Figure 4A, *p* = 0.67 and *p* = 0.076, respectively, Mann–Whitney *U*-test). As mentioned earlier (19), this type of analysis is only possible within the initial period of SE. For quantification of the entire SE, spike, and spectral analyses were extended to 6 h. While there was no difference in the number of spikes per minute during this period (*p* = 0.11, Mann–Whitney *U*-test), the normalized γ band power was significantly reduced in IPW-5371- vs. vehicle-treated kainate mice (Figures 4B,C, *p* = 0.037, Mann–Whitney *U*-test). These data indicated that TGFßR1 kinase inhibition interferes with acute seizure activity following kainate administration, specifically attenuating high-frequency activity in the γ range. The duration of the subsequent seizure-free (latent) phase was not influenced by IPW-5371 treatment (Figure 4A, right graph). Spontaneous generalized seizures (Figure 4D) were seen in 92.3% (12 of 13) of vehicle-treated and 85.7% (6 of 7) of IPW-5371-treated kainate mice (*p* = 0.73, log-rank test). As shown in Figure 4E (left graph), the total number of spontaneous generalized seizures during the 4 weeks of recording hardly differed between the conditions (*p* = 0.32, Mann–Whitney *U*-test). Likewise, the duration of individual generalized seizures was not different between the experimental groups (Figure 4E, right graph, *p* = 0.6, independent samples *t*-test). Importantly, during the entire recording period, the number of epileptic spikes per hour was significantly lower in the IPW-5371 group, indicating reduced total (e.g., ictal + interictal) activity (Figure 4F, *p* = 0.023, Mann–Whitney *U*-test). This finding was, however, not reflected by spectral analysis, since no difference was found in any of the frequency bands (data not shown). To assess a potential influence of the surgical procedure itself on seizure activity, brain activity was continuously EEG monitored for 9 days in a group of control mice that had received intracortical injection of 70 nl sterile NaCl (0.9%) instead of kainate. No seizures could be detected under these conditions (*n* = 6, data not shown).

**Figure 5:**
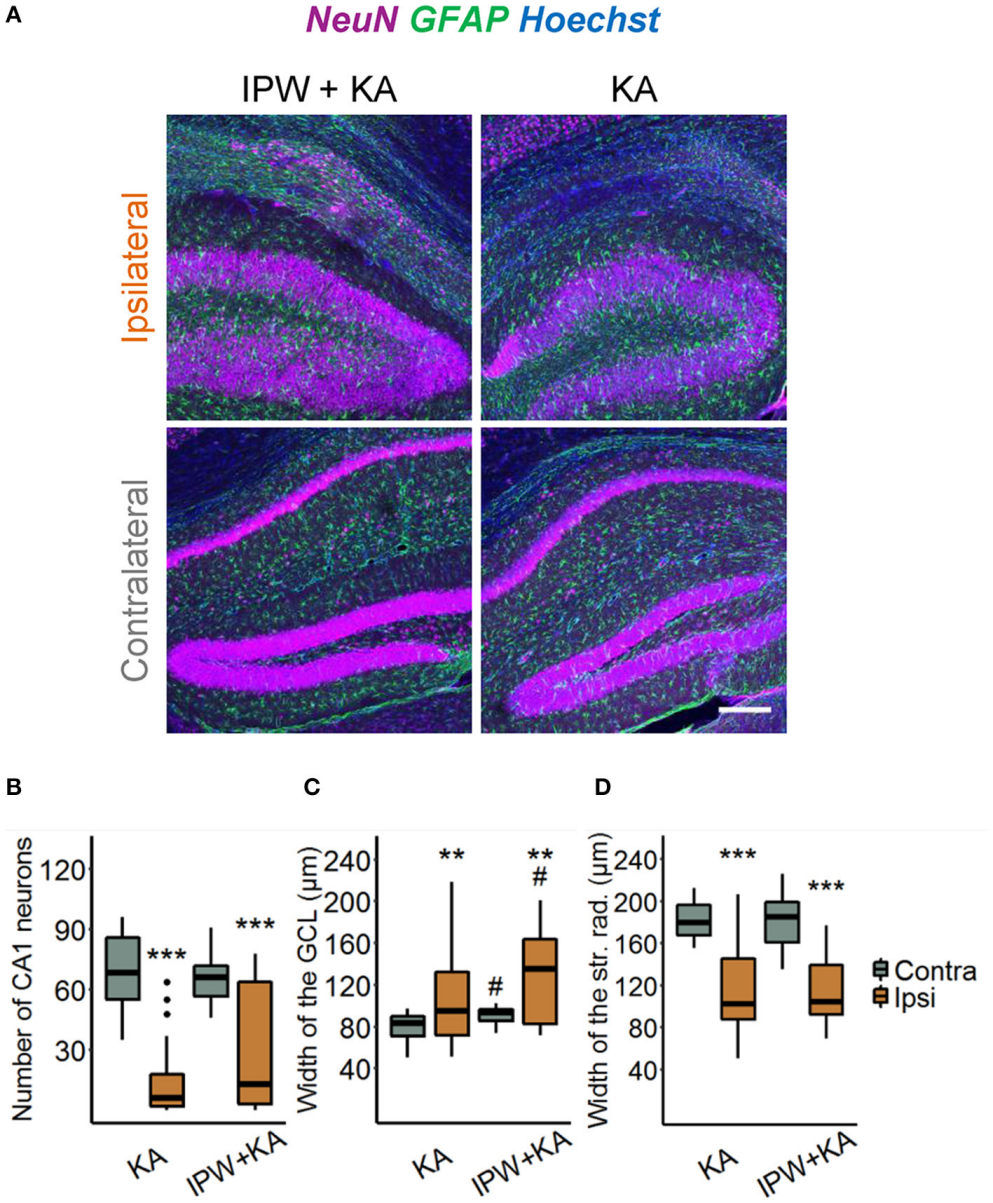
IPW-5371 treatment has no effect on the development of HS in experimental TLE. **(A)** Representative maximum intensity projections of combined NeuN (magenta), GFAP (green), and Hoechst (blue) staining in ipsi- and contralateral hippocampal slices from IPW-5371- and vehicle-treated kainate mice 1 month after kainate injection. Scale bar: 200 μm. Hippocampi of both IPW-5371- and vehicle-treated kainate mice displayed similar **(B)** pyramidal cell loss in the CA1 region, **(C)** GCD in dentate gyrus, and **(D)** shrinkage of the CA1 *stratum radiatum*. Data display box plots representing median and quartiles. ***p < 0.001 vs. contralateral, **p < 0.01, #*p* < 0.05 vs. control (two-way ANOVA). *N* = 29–31 slices from six animals/condition. CA, cornu ammonis; GCL, granule cell layer; str. rad., *stratum radiatum*.

We next explored the effect of IPW-5371 treatment on the development of HS. Hippocampal slices were stained with antibodies directed against NeuN, GFAP, and Hoechst 4 weeks after epilepsy induction (Figure 5A). Three hallmarks of HS—degeneration of CA1 pyramidal neurons, shrinkage of the CA1 region, and GCD—were evaluated. The extent of neurodegeneration was determined by counting the number of NeuN-positive cells in an area of 360 × 120 × 40 μm^3^ within the CA1 *stratum pyramidale* underneath the injection side and at the same position on the contralateral side. The data show that the ipsilateral loss of NeuN-positive cells was similar in IPW-5371- vs. vehicle-treated kainate mice (19.69 ± 92.42 vs. 8.76 ± 23.35% of contralateral; *p* < 0.001, two-way ANOVA, Figure 5B). Likewise, neither GCD (148.69 ± 61.71 vs. 114.49 ± 73.69% of contralateral, *p* = 0.0013, two-way ANOVA) nor CA1 shrinkage (56.49 ± 25.41 vs. 56.86 ± 32.17% of contralateral, *p* < 0.001, two-way ANOVA) was attenuated by IPW-5371 treatment (Figures 5C,D). Unexpectedly, kainate mice pretreated with IPW-5371 displayed increased GCD compared with vehicle-treated kainate mice both ipsi- and contralaterally (*p* = 0.014, two-way ANOVA). Taken together, these data reveal that inhibition of the TGFβR1/ALK5 pathway with IPW-5371 slightly reduces acute and chronic kainate-induced epileptiform activity but does not prevent the development of HS.

## Discussion

Our data confirm that leakage of albumin through a compromised BBB represents an early event and possibly one of the causative factors in epileptogenesis. Indeed, prominent albumin IR is observed in the brain parenchyma as early as 4 h after SE induction, preceding most of the epilepsy-associated histopathological alterations in our experimental model (14). This result is not surprising, as it is known from various animal models of seizures and epilepsy that BBB opening occurs within a few minutes after seizure induction [for review, see (27, 29)]. Although previous work concluded that BBB leakage in epileptogenesis is transient and lasts only a few days, more recent studies observed the dysfunction also in chronic human and experimental epilepsy, indicating that it may also contribute to the progression of the disorder (5, 26, 29–33). In agreement with this view, we detected albumin extravasation also 5 days and 3 months after SE, triggered by intracortical kainate injection (26). A surprising finding of the present study was the relatively high hippocampal albumin extravasation in sham-injected mice. Apparently, in this model, initial BBB leakage is not solely evoked by kainate-induced seizure activity but may also be caused by the injection itself. Because in this model damage to the hippocampus is avoided by intracortical injections, the phenomenon may arise from local tissue pressure changes and/or inflammatory processes evoked by the injected saline. The fact that extravasated albumin was only transiently seen in sham controls indicates that seizure activity is a prerequisite for long-lasting BBB opening. On the other hand, these control experiments provide evidence that transient albumin extravasation is not sufficient to cause neuronal hyperactivity or neuronal damage, since we have never observed seizures or histopathological changes in sham-injected mice.

A number of reports have proposed that extravasated albumin exerts its epileptogenic effects by altering essential astrocytic functions, such as their ability to buffer K^+^ and glutamate (11, 27, 34). Mechanistically, TGFβ receptor-mediated albumin uptake into astrocytes was proposed to mediate changes in gene expression responsible for these functional alterations (6, 8, 9, 15). However, some studies reported proepileptic effects of extravasated or injected albumin in the absence of astrocytic albumin IR, raising the question of whether uptake is really required for the albumin effects (13, 35). Interestingly, despite the lack of astrocytic albumin, Bankstahl and colleagues observed reduced GFAP and AQP4 IR in albumin-positive hippocampal regions during the early phase of pilocarpine-triggered epileptogenesis, showing that extravasated albumin can indeed influence astrocyte function without being taken up (35). Therefore, the lack of astrocytic albumin uptake found in our model does not exclude the possibility that albumin influences epileptogenesis *via* these cells.

Brain exposure to serum albumin impedes extracellular K^+^ ([K^+^]_o_) buffering by reducing the expression of astrocytic inward rectifying K^+^ channels (Kir 4.1) and gap junction proteins (6, 11, 15). According to the spatial K^+^ buffering concept, excessive extracellular [K^+^]_o_ released during neuronal activity is passively taken up by astrocytes through Kir4.1 channels, and then redistributed through the gap junction-coupled astrocytic network to be released at regions of lower [K^+^]_o_ (36). Consequently, loss of Kir4.1 expression or astrocytic coupling would result in accumulation of [K^+^]_o_, neuronal depolarization, and a lowered threshold for seizure generation. We have previously demonstrated Kir4.1 downregulation in chronic human TLE (37, 38) as well as reduced astrocytic coupling and impaired K^+^ clearance 4 h after SE induction in the intracortical kainate injection model of TLE (14). Since strong albumin extravasation was also found at this time point, it was reasonable to assume that albumin mediated the uncoupling. Our experiments with IPW-5371 do not support this hypothesis, although there is still the possibility that albumin affects coupling *via* a TGFβ-independent pathway. Moreover, because we did not examine Kir4.1 expression in this study, we cannot rule out that impaired K^+^ buffering in this model is mediated, at least in part, by albumin-induced downregulation of Kir4.1. Indeed, reduction of Kir4.1 expression may occur already 2 h after intracerebroventricular albumin injection (13), while downregulation of gap junction proteins and reduced interastrocytic coupling were demonstrated 24 h following exposure to albumin (11, 15). Disruption of astrocytic coupling in epileptogenesis is likely determined by different mechanisms at different time points during and after SE, and extravasated albumin may be involved at later time points of epileptogenesis.

Several studies provided convincing evidence that BBB leakiness contributes to epileptogenesis through albumin extravasation and activation of the TGFβ pathway (6, 8, 9). This work revealed that albumin and TGFβR1 possess similar proepileptic effects that are prevented by TGFβ pathway inhibition (9, 16). Our results show that early TGFβR1 inhibition only marginally affects the development of TLE, implying that BBB disruption and albumin extravasation over longer periods are required to crucially influence the process. Indeed, experimental opening of the BBB or albumin infusion over days was necessary to induce spontaneous seizures (6, 9, 10), while transient (short-term) hippocampal exposure to albumin, evoked by a single intracerebroventricular injection, was not sufficient to trigger seizures (13). According to its published pharmacokinetics, IPW-5371 effectively inhibits TGFβR1/ALK5 signaling for about 24 h (17). Future experiments are needed to reveal whether long-term inhibition (e.g., over the entire latency period, which lasts on average 5 days in this model) would completely suppress epileptogenesis. It must be stressed, however, that our data do not provide information about whether TGFβ signaling is activated by extravasated albumin and whether its proepileptic action is caused by changes in astrocytic function. Not only astrocytes but virtually all cell types in the brain, including neurons, microglia, and endothelial cells, produce TGFβ and possess TGFβ receptors (39). Our immunostaining indicated neuronal albumin uptake (at 24 h, but not yet 4 h post kainate, Supplementary Figure 1), an observation that matches several other studies (5, 13, 15, 40). Therefore, it would also be possible that albumin directly affects neuronal activity *via* TGFβ signaling. Independent of this, extravasated albumin seems to affect neuronal excitability without influencing the strong histopathological alterations characteristic of HS. This result is consistent with previous research that found no evidence for albumin-induced neurodegeneration (10, 13, 34, 35). Unexpectedly, we observed even more pronounced GCD in IPW-5371-pretreated mice, both ipsi- and contralaterally. How TGFβR1 signaling regulates seizure-induced GCD remains to be elucidated in future experiments.

## Conclusion

We show, in a mouse model closely resembling human TLE with HS, that albumin extravasation into the brain parenchyma arises very soon after SE induction. At this early stage, albumin is not yet taken up by astrocytes and uncoupling is not the result of albumin-stimulated TGFβR1/ALK5 signaling. Whether the latter is involved in the complete loss of coupling seen in chronic experimental and human epilepsy remains to be investigated. Inhibition of TGFβ signaling during the first hours of kainate-induced SE only slightly affected epileptogenesis in our TLE model, suggesting that longer-lasting albumin extravasation is necessary to critically alter the pathological process. Our results provide new insights into the role of BBB dysfunction and the development of TLE, which may help in identifying new targets for antiepileptogenic strategies.

## Data Availability Statement

The original contributions presented in the study are included in the article/Supplementary Material, further inquiries can be directed to the corresponding author/s.

## Ethics Statement

The animal study was reviewed and approved by North Rhine–Westphalia State Agency for Nature, Environment and Consumer Protection (approval number 84-02.04.2015.A393).

## Author Contributions

CS and PB designed and supervised the experiments, LH and PB performed and analyzed the experiments. All authors wrote the manuscript, contributed to the article, and approved the submitted version.

## Conflict of Interest

The authors declare that the research was conducted in the absence of any commercial or financial relationships that could be construed as a potential conflict of interest.
