# Supplementary material for: Initiation of Experimental Temporal Lobe Epilepsy by Early Astrocyte Uncoupling Is Independent of TGFβR1/ALK5 Signaling
Source: Front Neurol. 2021 May 7;12:660591. doi: 10.3389/fneur.2021.660591 (PMC8137820; doi:10.3389/fneur.2021.660591)
Supplement: Supplementary file 1 [file Data_Sheet_1.PDF]

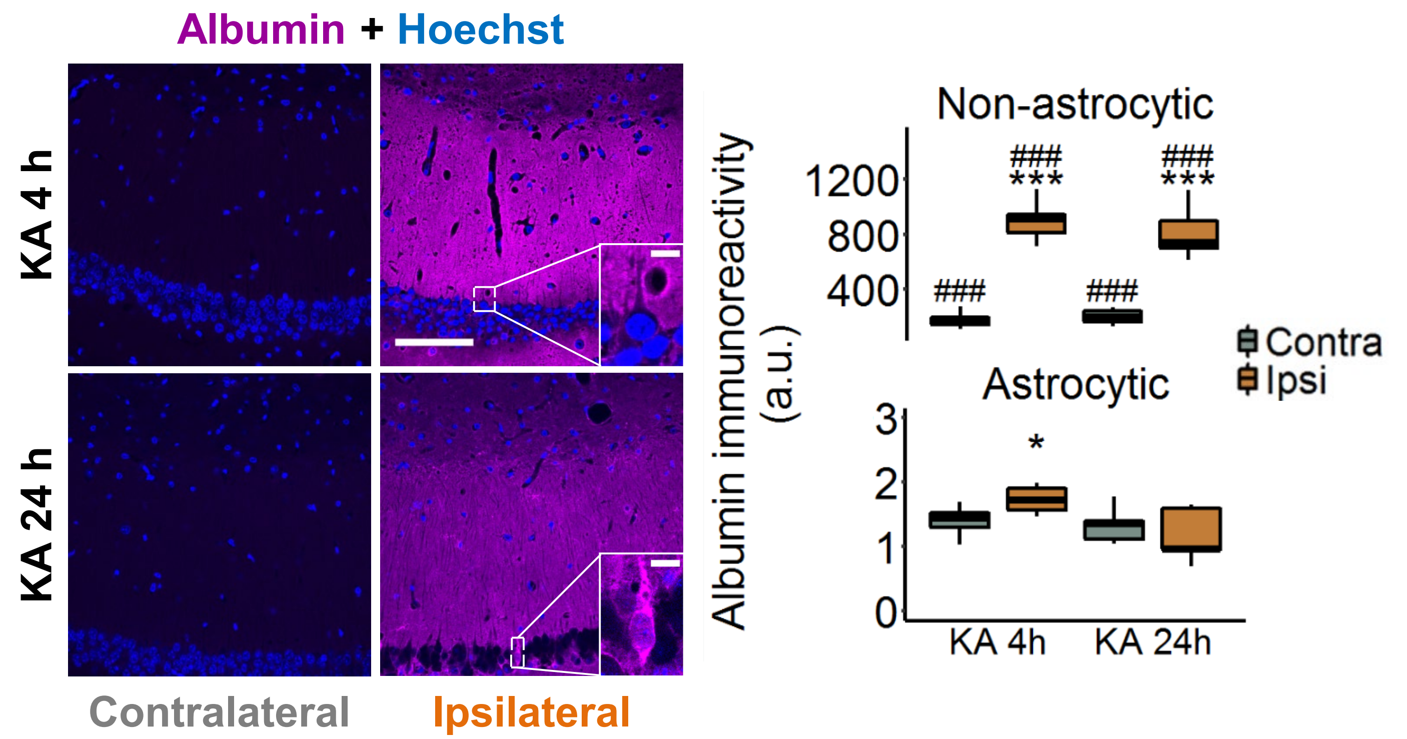

Supplementary Figure S1: Albumin is largely absent in astrocytes at 4 and 24 h post kainate, but appears to be taken up into ipsilateral CA1 pyramidal neurons, 24 h post kainate injection *(left panel, bottom right).* Scale bar: 100 µm (insets: 10 µm). * *P < 0.05,* *** *P < 0.001* vs contralateral, ### *P < 0.001* vs astrocytic (*Two-way ANOVA)*.
